# Dose-staged Gamma Knife radiosurgery for meningiomas: A retrospective study in a single center

**DOI:** 10.3389/fneur.2022.893480

**Published:** 2022-10-13

**Authors:** Xiu Gong, Jianbo Ding, Jonathan P. S. Knisely, Enmin Wang, Li Pan, Binjiang Wang, Nan Zhang, Hanfeng Wu, Jiazhong Dai, Tonggang Yu, Xuqun Tang

**Affiliations:** ^1^Department of Neurosurgery, Huashan Hospital, Shanghai Medical College, Fudan University, Shanghai, China; ^2^Department of Neurosurgery, Shanghai Gamma Hospital, Shanghai, China; ^3^Department of Neurosurgery, Gamma Knife Center of Huashan Hospital, Shanghai, China; ^4^Department of Radiation Oncology, Weill Cornell Medicine and New York-Presbyterian Hospital, New York, NY, United States

**Keywords:** meningioma, staged radiosurgery, Gamma Knife, dose-staged GKRS, two-stage

## Abstract

**Objective:**

This study aimed to study the efficiency and safety of a dose-staged Gamma Knife radiosurgery strategy for large meningiomas or meningiomas close to important nerve structures.

**Methods:**

This study evaluates the outcome of a prospectively accrued series of 71 consecutive patients with meningiomas treated with staged dose-fractionated Gamma Knife radiosurgery. The average peripheral doses for the first and second fractions were 9.0 ± 0.9 Gy (8–12 Gy) and 8.6 ± 0.7 Gy (range, 7–10 Gy), respectively. The interval between fractions was 6.1 ± 1.9 months (range, 3–12 months). The median follow-up time was 36 months (12–96 months).

**Results:**

During the follow-up period after the second fraction, 97.2% achieved tumor control in our series. A total of 2 patients exhibited local recurrence at 30 and 60 months after the second fraction, respectively. No treatment-related complications or new long-term neurological dysfunctions were reported. MRIs observed slightly or moderately increased peritumoral edema in six patients, but no specific neurological complaints are attributed to this finding.

**Conclusion:**

This study investigates the efficiency and safety of dose-staged Gamma Knife radiosurgery as an alternative option for meningiomas that were large in volume, adjacent to crucial structures, or in patients with contraindications to craniotomy.

## Introduction

Meningioma is a common primary intracranial tumor, with 53.7% of all primary non-malignant brain tumors (1). If total resection can be achieved without causing neurological damage, microsurgical resection is the best treatment option. However, when meningiomas are located close to critical structures, morbidity rates are increased (2). In addition, some patients have contraindications to general anesthesia and craniotomy. Stereotactic radiosurgery is the primary treatment for WHO grade 1 meningioma when the lesion volume is small and the imaging features are typical, with an 85–100% tumor control rate achieved at 5 years of treatment (3, 4). Conventional radiotherapy is mainly used as adjuvant therapy after surgery for WHO grades 2 and 3 meningiomas (3–5). For meningiomas patients who would not tolerate craniotomy, the single-fraction Gamma Knife treatment with a peripheral dose of 12–14 Gy in one session has been proven effective. However, the treatment only works with tumors with an average diameter of <3–3.5 cm to avoid serious radiation-induced toxicity (6, 7). Single-fraction Gamma Knife radiosurgery (GKRS) is limited by the radiation tolerance of critical functional areas, such as the optic nerves or chiasm, which cannot deliver the effective radiation dose to tumors close to these structures (8, 9). The previous studies on superior alternative radiation treatment for patients with surgical contraindications with large meningiomas or meningiomas close to important nerve structures are limited.

Hypofractionated stereotactic radiosurgery, treating a lesion in 2–5 fractions of SRS, can potentially provide the ability to treat large tumors at adequate tumor control and acceptable toxicity (8, 10). However, details on treating large meningiomas with fractionated Gamma Knife radiosurgery are rarely reported. Limited studies have presented early results for the mode of volume-fractionated staged radiosurgery to treat large skull base meningiomas (11–14). This mode divides a large lesion into two parts according to the volume. Each treatment adopts a dose similar to the single-fraction Gamma Knife to treat different tumor parts, with inter-fraction intervals of 3–9 Months. This study developed a dose-staged Gamma Knife radiosurgery strategy (dose-staged GKRS) for patients with surgical contraindications suffering from large meningiomas or meningiomas close to critical nerve structures. The whole target was covered with a lower dose, with an inter-fraction interval of 6 months in every fraction. This study demonstrates the experience obtained in a relatively large series of meningiomas treated with dose-staged GKRS.

## Materials and methods

### Inclusion criteria and exclusion criteria

Meningiomas patients that meet the following criteria were included in this study: the volume of meningiomas without previous surgery is greater or equal to 10 cc; the volume of residual or recurrent meningiomas after surgery was greater or equal to 10 cc; meningiomas are located near the optic nerve pathway, and the patient has useful vision regardless of the tumor volume or whether or not surgery has been performed.

Patients with WHO grade 2–3 meningiomas or who had progression of their tumor after prior irradiation were excluded.

### Patient profile and diagnostic criteria

A total of 71 consecutive patients were included in the retrospective, all with WHO grade 1 meningioma treated with dose-staged GKRS at Shanghai Gamma Hospital (Gamma Knife Center of Huashan Hospital) between June 2013 and March 2020. The characteristics of all patients are listed in Table 1. These patients served as a prospectively accrued consecutive series, consisting of 14 (19.7%) men and 57 (80.3%) women, with an average age of 52.3 ± 11.2 years (range, 31–85). 28 (39.4%) patients had undergone surgical resection, and the meningioma diagnosis was confirmed by histopathology. For 43 (60.6%) patients, radiosurgery was the initial treatment, and the diagnosis of meningioma was based on MRI and CT characteristics. The mean initial tumor volume of all cases in this series was 12.7 ± 9.3 cm^3^ (range, 0.6–41.1 cm^3^). To analyze the size effect of treated lesions on the volumetric outcome, we divided all cases into three groups (<8 cc for 29 cases, 8–20 cc for 28 cases, >20 cc for 14 cases). Besides, the tumors were located in the skull base (59 cases), parasagittal sinus (9 cases), lateral ventricle (1 case), pineal region (1 case), and tentorium (1 case).

**Table 1:** Characteristics of the 71 patients in this series.

**Sex (*n*, %)**	
Male	14 (19.7%)
Female	57 (80.3%)
Age in years (mean ± SD, range)	52.3 ± 11.2 (31–85)
**Primary or postoperative treatment (*n*, %)**	
Primary	43 (60.6%)
Postoperative	28 (39.4%)
**Initial tumor volume (*n*, %)**	
<8 cc	29 (40.8%)
8–20 cc	28 (39.4%)
>20 cc	14 (19.7%)
**Tumor location (*n*, %)**	
Skull base	59 (83.1%)
Parasagittal sinus	9 (12.7%)
Lateral ventricle	1(1.4%)
Pineal region	1(1.4%)
Tentorium	1(1.4%)
Interval in months between radiosurgery stages (mean ± SD, range)	6.1 ± 1.9 (3–12)
Peripheral dose (Gy) for first treatment (mean ± SD, range)	9.0 ± 0.9 (8–13.5)
Peripheral dose (Gy) for second treatment (mean ± SD, range)	8.6 ± 0.7 (7–10)

The WHO Classification of Tumors of the Central Nervous System classifies meningiomas into three grades and 15 subtypes. WHO grade 1 meningiomas consist of benign tumors with nine subtypes (15). Before Gamma Knife radiosurgery treatment, the patients were evaluated by a clinical examination, such as a detailed neurological examination, thin-slice, contrast-enhanced MRI, and high-resolution computed tomography (CT). A multidisciplinary team of neurosurgeons, neuroradiologists, and radiation oncologists evaluated each patient for treatment eligibility. For patients without histopathology, CT and MRI features were evaluated separately by two qualified neuroradiologists following the EANO guidelines by T1WI signal strength, T2WI signal strength, and the degree of peritumoral edema, respectively, and the degree of enhancement of the tumors (5).

### Dose-staged Gamma Knife radiosurgery technique

Dose-staged Gamma Knife radiosurgery treatments were conducted under local anesthesia using a Leksell Gamma Knife model Perfexion (before January 2019) and ICON (from February 2019) at Shanghai Gamma Hospital, Gamma Knife Center of Huashan Hospital. Gadolinium-enhanced images of T1-weighted, T2-weighted, and FLAIR sequences were collected from each patient for pretreatment localization using a 1.5-T MR imaging system (Signa Excite, GE, USA). The tumor and adjacent critical structure delineation, dose prescription, and planning were conducted by a neurosurgeon, radiation oncologist, and radiation physicist. Dose planning was performed using MR images mentioned above that were exported to the Leksell GammaPlan software (version 10.0, Elekta Instruments AB, Stockholm, Sweden). The peripheral dose was administered to cover the gross tumor volume with no additional margin. An independent central physician evaluates every experiment plan before treatment is conducted.

### Prescription dose and interval

**Figure 1:**
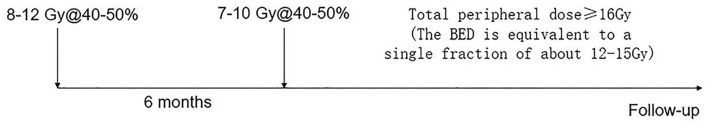
The treatment protocol for two-staged radiosurgery for meningiomas in this series is ~6-month interval between fractions.

This study used a two-stage treatment mode for all patients. The prescription dose for each fraction depended on the tumor volume and dose tolerance of the near nerves and brain structures. The biological equivalent dose (BED) of normal brain tissue and the tumor was calculated for α/β = 3 Gy. In theory, the total dose for individual patients was calculated and divided into two-staged treatment. The average peripheral doses for the first and second fractions were 9.0 ± 0.9 Gy (range, 8–12 Gy) and 8.6 ± 0.7 Gy (range, 7–10 Gy), respectively (Table 1). The prescription isodose line for each fraction was between 40 and 50% and did not require to be equal for the two stages. The interval between stages was 6 months. Still, the date of the second stage of treatment was altered for follow-up imaging and side effects after the first stage of treatment (Figure 1).

### Follow-up evaluations and toxicity

The first follow-up was carried out for each patient 3–6 months after the first stage of the treatment by interviewing the patients, examinations, and MR imaging to assess changes in patients' clinical symptoms and tumor volumes and determine the second stage administration date. Patients who exhibit satisfactory tumor control would receive regular clinical and MRI follow-up every 6 months after the second stage of treatment during the first year, annually for the next 2 years, and once every 2 years (Figure 1). The tumor volumes at each treatment stage and follow-up were calculated and compared using the patient's MRI data in the Leksell GammaPlan software. The tumor control is evaluated based on the change in tumor volume. Tumor control is considered good when the tumor volume decreases. If the tumor volume increases by more than 20%, an analysis of the causes is required, and salvage treatment is conducted when necessary.

All toxicities were scored according to version 5.0 of the National Cancer Institute Common Terminology Criteria for Adverse Events (16).

### Statistical analysis

The SPSS software, version 25.0 (SPSS, Chicago, IL, USA), was used for statistical analysis. Values are presented as mean ± standard deviation data with normal distribution or median and interquartile range for data that were abnormally distributed for continuous variables and number (%) for categorical variables. For multi-group comparisons, *p* values were derived from one-way ANOVA. For all comparisons, *p* < 0.05 was considered statistically significant.

## Results

### Tumor control

**Figure 2:**
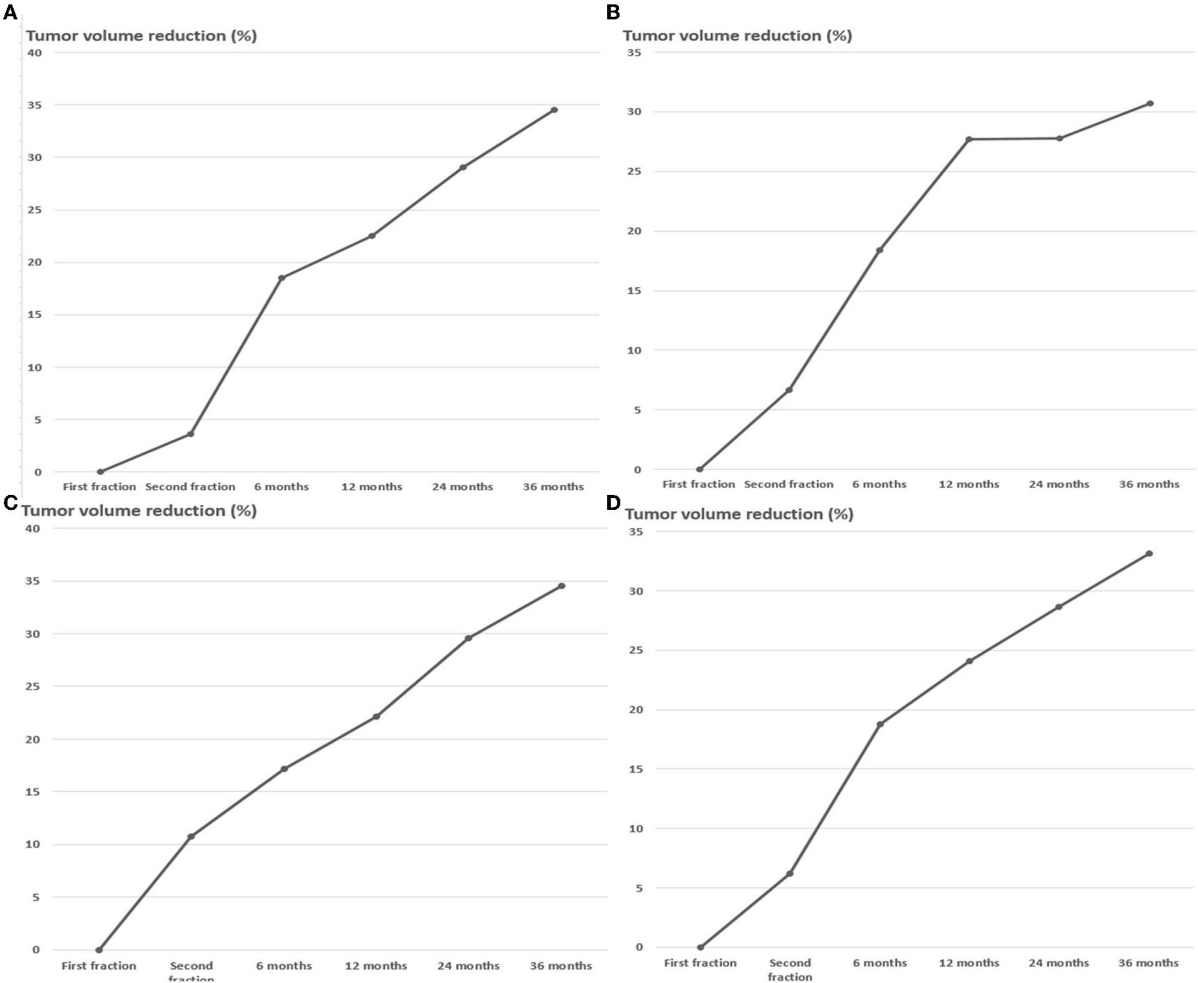
Tumor volume trends for meningiomas of different initial volume after dose-staged radiosurgery **(A)** <8 cc group, **(B)** 8–20 cc group, **(C)** >20 cc group, **(D)** Combined group.

**Figure 3:**
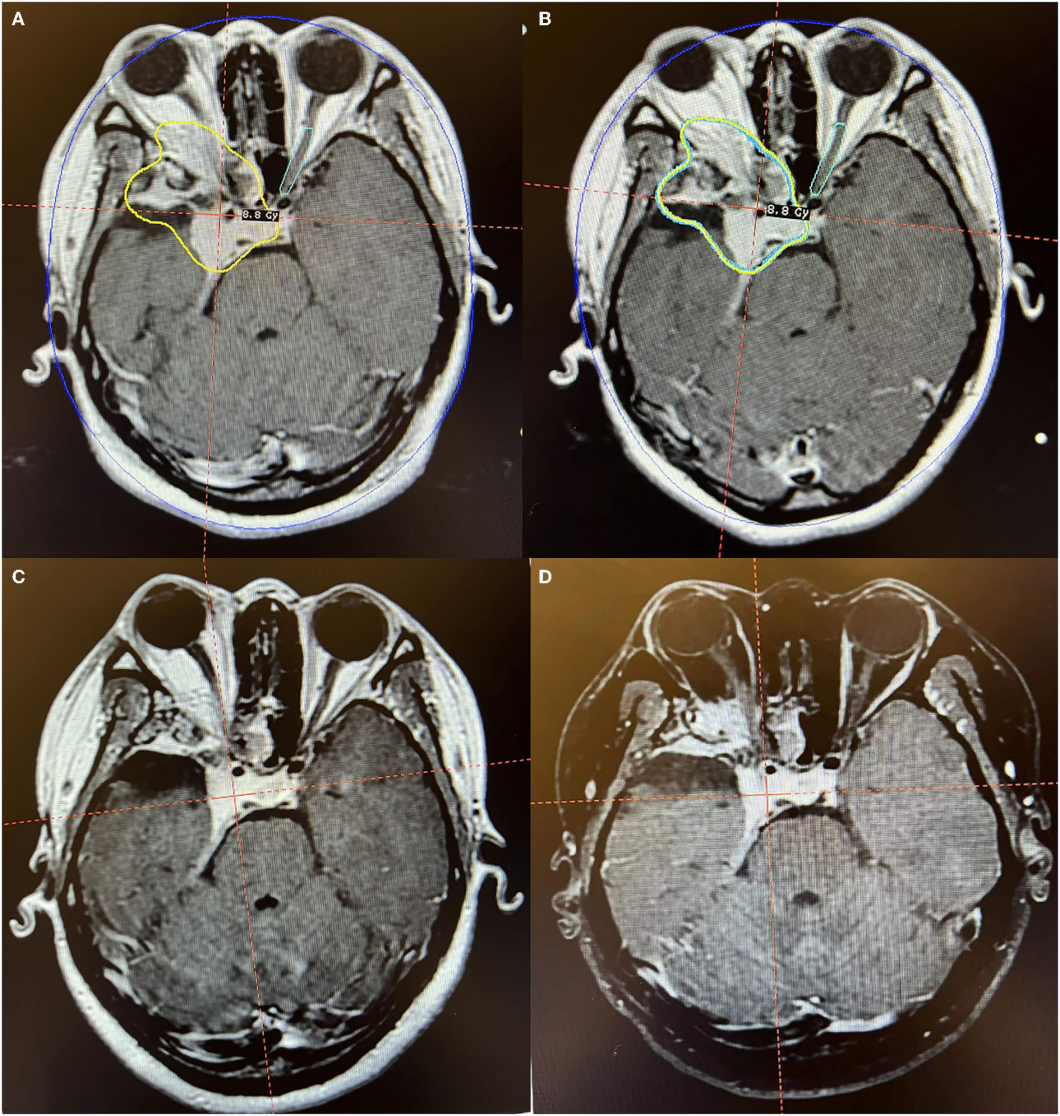
Axial MR imaging illustrating a huge postoperative residual orbitocranial meningioma post-operation treated with Gamma Knife radiosurgery. **(A)** The prescription dose planning MRI for the first stage of Gamma Knife radiosurgery showing that the tumor margin was covered by 8.8 Gy (45% isodose surface). **(B)** The prescription dose planning MRI for the second stage of Gamma Knife radiosurgery demonstrated that the tumor decreased by 9.8% and was treated with the same dose as the first stage. **(C)** MRI obtained 12 months after radiosurgery presented a decrease of 27.3% in tumor size. **(D)** MRI obtained 36 months after radiosurgery manifested that the tumor is under stable control. The patient's right eye was blind before radiosurgery. After Gamma Knife treatment, there were no obvious adverse reactions, and the vision of the left eye was unchanged.

**Figure 4:**
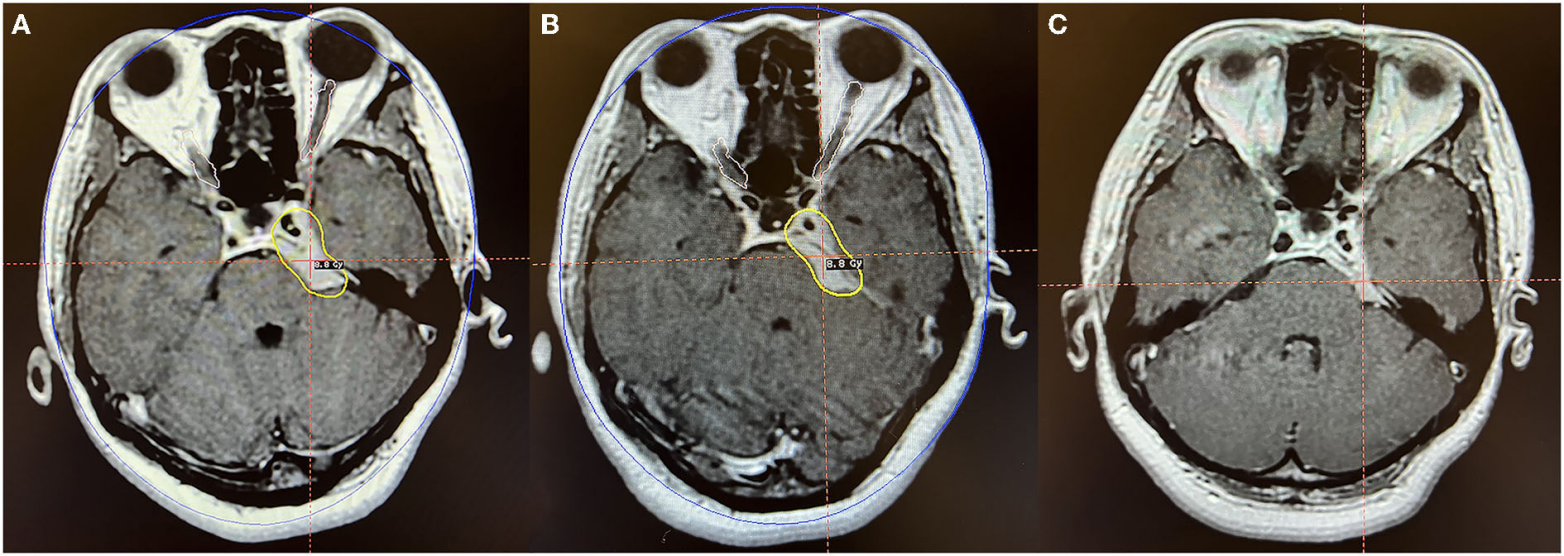
Axial MR imaging illustrating a petroclival middle-posterior communicating meningioma treated with Gamma Knife radiosurgery. **(A)** The prescription dose planning MRI for the first stage of Gamma Knife radiosurgery displaying that the tumor margin was covered by 8.8 Gy (45% isodose surface), and the tumor volume was 7.0 cc. **(B)** In the second fraction, the tumor was treated with the same dose as the first stage, and the tumor volume was 6.1 cc. **(C)** MRI obtained 24 months after radiosurgery indicated that the tumor volume is 4.7 cc, decreased by 33.0% compared with the pre-radiosurgery volume. No obvious adverse reactions were observed.

**Figure 5:**
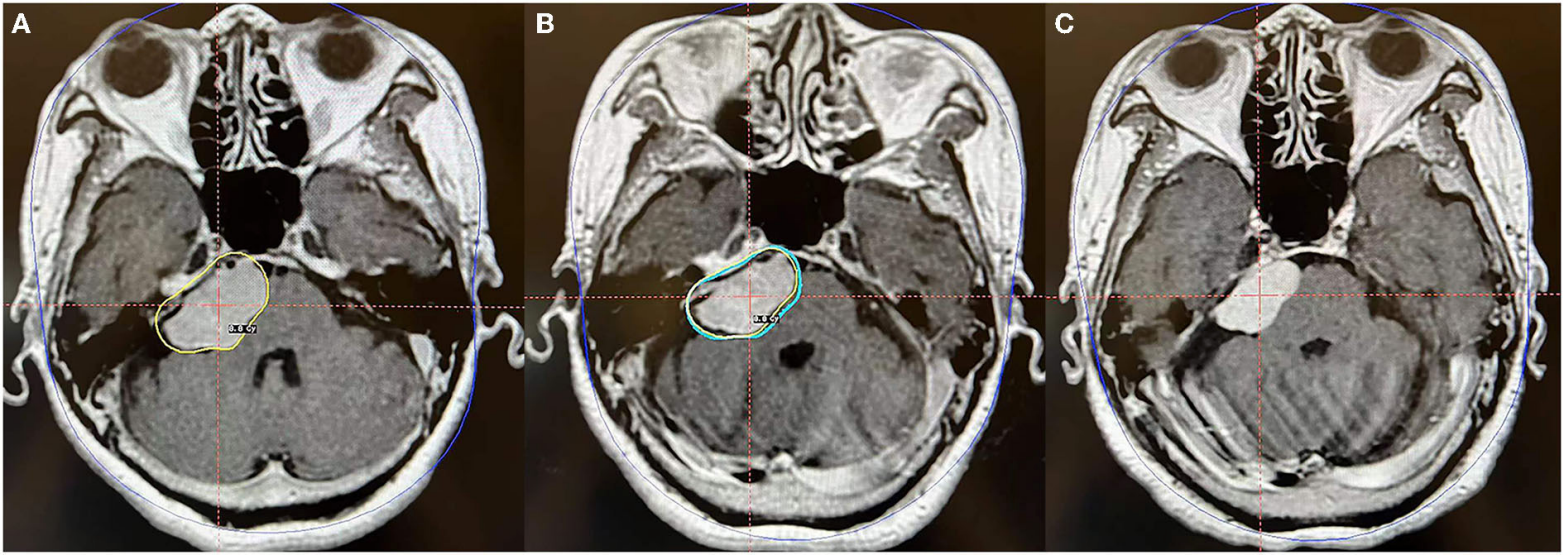
Axial MR imaging illustrating a posterior petrosal meningioma treated with Gamma Knife radiosurgery. **(A)** The prescription dose planning MRI for the first stage of Gamma Knife radiosurgery presenting that the tumor margin was covered by 8.8 Gy (45% isodose surface), and the tumor volume was 11.0 cc. **(B)** In the second fraction, the tumor was treated repeatedly with the same precision dose as in the first stage, and the tumor volume was 8.4 cc. **(C)** MRI obtained 24 months after radiosurgery revealed that the tumor volume is 6.1 cc and is decreased by 44.5% compared with that pre-radiosurgery. No obvious adverse reactions were observed.

By the end of November 2021, the median follow-up time for this study was 36 months (range, 12–96 months). The average tumor volume between the first and second stages was decreased by 6.2 ± 13.5%. However, the tumor volume of 17 (23.9%) cases increased by 11.1 ± 6.9%. In the other 54 (76.0%) cases, the mean tumor volume decreased by 11.3 ± 8.5%. Most patients were followed-up through November 2021, but some patients missed one or several scheduled follow-up MRI scans. Recurrent cases were excluded from further follow-up. The MRI follow-up data were obtained from 65 (91.5%), 55 (77.5%), 48 (67.6%), and 38 (53.5%) individuals after 6, 12, 24, and 36 months of second-stage treatment, respectively. The follow-up data from 6 months after the second treatment depicted that the volume of all lesions decreased by 18.7 ± 14.5%. The follow-up data at 12, 24, and 36 months demonstrated that the tumor shrank by 24.1 ± 14.5, 28.7 ± 15.0, and 33.1 ± 14.5%, respectively (Figures 2–5). The percentage reduction in the volume of the three subgroups grouped according to the initial volume has no significant difference in each period after treatment (Figure 2, Table 2).

**Table 2:** Mean percentage reduction in volume for meningiomas with different initial volume after dose-staged Gamma Knife (%).

**Time period**	**Total, *n* = 71**	**<8 cc, *n* = 29**	**8–12 cc, *n* = 28**	**>12 cc, *n* = 14**	***P***
Second fraction	5.96	2.97	6.64	10.79	0.263
6 months	16.24	16.78	15.29	17.14	0.928
12 months	23	24.12	22.18	22.12	0.467
24 months	28.19	27.81	27.82	29.61	0.948
36 months	31.16	36.27	33.3	34.52	0.751

After the second stage and in follow-up, the overall tumor control rate was 97.2% (69/71). Local recurrences occurred in 2 cases, with the time of recurrences at 30 and 60 months after the second stage of treatment. Those cases had not undergone previous surgery before Gamma Knife. One case with a sellar region meningioma reported vision loss 60 months after the second stage of radiosurgery. MRI showed the tumor recurred from its original position, and the patient immediately received salvage radiosurgery. After the surgery, the tumor was reduced by 23.3%, and the patient's visuals were restored gradually from 3 months after treatment. Another patient was diagnosed with tumor recurrence on a surveillance MRI 30 months after the second stage of treatment and received further surgery, confirming the diagnosis of WHO grade 1 meningioma.

### Clinical response and toxicity

Before radiosurgery, 35 (49.3%) patients had neurological symptoms, and 36 (50.7%) patients were initially asymptomatic. No obvious aggravation was reported in specific symptoms in all patients after the first stage of treatment. However, MRI showed signs of temporary tumor tissue swelling (reduction of central area enhancement and slight expansion of volume) and mild edema of peripheral brain tissue in 13 (18.3%) cases. The second stage was delayed for 1–3 months to reduce the radiation-related risk and improve the tolerance of these patients to radiosurgery. After the second stage, among 35 patients with neurological dysfunction, 11 patients had improvement and remained stable, and the other 24 displayed no aggravation of symptoms. A total of 15 (21.2%) patients reported nonspecific headache or dizziness within 6 months after the second stage of treatment. Still, the headache was mild and had little effect on daily life, and gradually disappeared after 1 month without special treatment. During the follow-up period, limited edema around the lesions was slightly increased in six cases; these tumors were located in the skull base (4 cases), parasagittal sinus (1 case), and lateral ventricle (1 case), respectively (Table 3). Still, the tumor size was stable or shrunk, and these patients reported no specific symptoms.

**Table 3:** Outcomes of clinical response and toxicity of dose-staged Gamma Knife for meningiomas in different location.

**Outcomes**	**Skull base, *n* = 59, (%)**	**Parasagittal sinus, *n* = 9, (%)**	**Lateral ventricle, *n* = 1, (%)**	**Pineal region, *n* = 1, (%)**	**Tentorium, *n* = 1, (%)**	**Total, *n* = 71, (%)**
Tumor control	57 (96.6)	9 (100)	1 (100)	1 (100)	1 (100)	69 (97.2)
Progression	2 (3.4)	0	0	0	0	2 (2.8)
GKRS related edema	4 (6.8)	1 (11.1)	1 (100)	0	0	6 (8.5)
Clinical progression	0	0	0	0	0	0
Headache	11 (18.6)	3 (33.3)	1 (100)	0	0	15 (21.1)

## Discussion

The meningioma treatment is widely dominated by local treatment, such as craniotomy or local radiosurgery. The classic single-fraction Gamma Knife radiosurgery has a therapeutic effect with a low incidence of toxic reactions for “suitable cases” and, in the long-term, can achieve tumor control rates similar to Simpson Grade 1 surgical resection (17, 18). However, “suitable cases” often denote small tumors far from the critical structures (e.g., optic nerve and brainstem). Some meningiomas patients have large lesions in clinical practice, but their physical conditions can poorly tolerate craniotomy. Other patients may present with tumors close to the optic nerve and other critical structures. Despite the small tumor, a conventional prescription dose may risk consequential radiation damage to the brain tissue or critical structures. These circumstances have limited the application of Gamma Knife radiosurgery and make it difficult for single-fraction Gamma Knife radiosurgery (19–22).

About 40 years ago, a staged Gamma Knife strategy was used to treat large AVMs and achieved positive results (23–26). In recent decades, staged Gamma Knife radiosurgery was used to treat brain metastases and meningiomas (11–14, 27–31). Unlike conventional radiotherapy, which requires daily treatments for 5–6 weeks, staged Gamma Knife radiosurgery has a long interval between two sessions. Staged Gamma Knife radiosurgery consists of two stages (11–14, 23–32): volume-staged Gamma Knife radiosurgery in which different fractions of the dose planning curves cover the different subvolumes of the target; and dose-staged Gamma Knife radiosurgery in which every fraction uses a lower dose to cover the whole target.

Few studies have reported the application of staged Gamma Knife radiosurgery for meningioma treatments, which all employed a volume-staged approach. The volume-staged approach divides the target into two or more parts, and the treatment volumes and doses of each stage differ. Iwai et al. reported 7 cases of skull base meningiomas treated with volume-staged Gamma Knife radiosurgery in 2001 (11). The volume of each treatment was 6.8–29.6 cm^3^ (mean 18.6 cm^3^), with an inter-fraction interval of 6 months and marginal doses of 8–12 Gy for each fraction. Six of seven patients achieved tumor growth control during the follow-up period (mean 39 months). The number of cases increased to 27 in 2019 with a similar treatment strategy. With an average follow-up of 84 months, only 25% of cases reported local tumor control failure, and 4% reported permanent radiation injury (12). In 2009, Haselsberger et al. reported 20 staged Gamma Knife radiosurgery cases for large meningiomas in critical locations (13). The volume treated in each session was 5.4–42.9 cm^3^ (median 19.0 cm^3^), the treatment interval was between 1 and 12 months (median: 6 months), and the median prescription dose with 45% isodose surface was 12 Gy (range: 10–25 Gy). During a median follow-up of 7.5 years, 90% of the patients achieved tumor control (25% tumor regression, 65% stable size). Su et al. reported 4 cases of large skull base meningioma surrounding the optical apparatus (14), for which treatment mode of 2–3 fractions was administered with intervals of 4–6 months. In stage 1, ~3/4 of the tumor volume far from the optic nerve (13.2 cm^3^, range: 3.9–54.7 cm^3^) was treated with a marginal dose of 13.5 Gy (range: 12–15 Gy). In stage 2, the upper portion of the tumor located close to the optic nerve was treated (4.3 cm^3^; range: 1.5–16.2 cm^3^), and the marginal dose was 9 Gy (range: 8–10 Gy). A 34–46% reduction in tumor volume was reported during a median follow-up period of 100.5 months. The efficiency and safety of the treatment are generally satisfactory.

Nevertheless, the number of cases in each series is relatively limited, and the approaches used in each stage vary to a great degree. For volume-staged radiosurgery, at the site of the junction between subvolumes, the center or sub-center of the tumor, the dose delivered to the meningioma is insufficient to control growth durably. Conversely, the dose delivered in 2 fractions to adjoining normal brain tissue may exceed its tolerance level. Experiment and clinical data cannot decide the best approach for fractionated radiosurgery for meningiomas. Detailed research is required to design safe and effective staged Gamma Knife radiosurgery meningioma plans that stabilize the relationship between a single prescription dose and treatment interval.

This study represents the first report of dose-staged Gamma Knife radiosurgery for meningiomas. Dose-staged Gamma Knife radiosurgery more closely fulfills tumor radiobiology's fundamental principles. Unlike volume-staged radiosurgery, the schedule of dose-staged Gamma Knife radiosurgery gives more stable radiation doses to the tumor. The contiguous normal brain tissue has a longer repair period and lowers integral doses of radiation; avoid exposing brain tissue at the sites where adjoining stages of volume-staged radiosurgery complement the high dose of radiation from each fraction. Seventy-one cases were included in our study, and compared with other published articles; this is the largest number of cases reported yet for staged Gamma Knife radiosurgery of meningiomas. It was found that after a median follow-up of 36 months, 97.2% (69/71) of the lesions were reduced or stable after treatment, and only two patients had a local failure. The therapeutic effect is satisfactory compared to previous literature listed in Table 4.

**Table 4:** List of studies regarding staged Gamma-Knife radiosurgery for meningioma.

**Authors & year**	**No. of cases**	**Fractionated mode**	**Prescribe dose & fractions**	**Interval (m)**	**Tumor control**	**Permanent radiation injury (%)**
Iwai et al. (11)	7	volume-staged	8–12 Gy × 2 f	6	86% at 39 m	0
Haselsberger et al. (13)	20	volume-staged	12 Gy × 2 f	6	90% at 7.5 y	0
Su et al. (14)	4	volume-staged	8–15 Gy × 2–3 f	4–6	100% at 100.5 m	0
Iwai et al. (12)	27	volume-staged	8–12 Gy × 2 f	3–9	75% at 84 m	4
Present study	71	dose-staged	9 Gy × 2 f	6	97.2% at 36 m	0

Leksell Gamma Knife model ICON can provide continuously fractionated radiotherapy treatment. A relatively fixed dose is usually implemented in 3–5 consecutive days (10, 33). This study used stage radiosurgery with a long interfraction interval instead of fractionated radiosurgery delivered on interval days. A slow response was observed in benign meningiomas to radiosurgery, where tumor tissue swelling and edema of peripheral brain tissue were reported from 3 to 6 months after radiosurgery (2, 34). Radiation-related adverse reactions after continuously delivered fractionated radiotherapy are unavoidable. Staged radiosurgery can adjust the treatment time and the dose for the second stage based on the treatment results of the first stage and the adverse side effects. After the first stage of treatment, 13 (18.3%) patients showed MRI signs of transient tumor tissue swelling and mild edema of peripheral brain tissue. Reports on the radiosurgery treatment of large meningiomas are limited, and the efficacy and biological mechanism of both continuously delivered fractionated radiotherapy and staged radiosurgery require further investigation.

In addition, 17 (23.9%) lesions had slightly increased by 11.1 ± 6.9% during the second stage of treatment. Although the degree of enlargement in these cases is small, it is considered a transient swelling of tumor tissue after radiosurgery instead of tumor progression. This is due to two aspects: firstly, WHO grade 1 meningioma normally progresses very slowly even without treatment; secondly, contrast-enhanced MRI showed that the enhancement of these tumors had slightly decreased. During the follow-up period of 6–36 months after the two stages of treatment, the lesion volumes gradually reduced to the proportion of the patients with no acute inflammatory changes.

This study suggests that dose-staged Gamma Knife radiosurgery has minimal adverse side effects and is well-tolerated for large meningiomas or those close to important nerve structures. The relatively longer interval of the two stages provided a sufficient “buffer period,” ensuring that patients with poor physical conditions could sustain the treatment plan. As a result, the tumor received a sufficient prescription dose, with only two lesions' recurrence at 30 and 60 months after radiosurgery. The tumor volume of the patients decreased by an average of more than 30% during the follow-up period of 36 months. Although 15 patients reported no headache or dizziness 6 months after the second stage of treatment, these symptoms were usually mild, had little effect on daily life, and gradually relieved after about 1 month without special treatment. After dose-staged Gamma Knife radiosurgery, the MRI detected edema around the tumor was slightly larger than that before Gamma Knife radiosurgery in six cases. Still, the tumor size was stable or reduced, with most patients showing no symptoms. No new neurological dysfunction related to radiosurgery was found in this study. Among the 35 patients with neurological dysfunction, 11 reported relief of symptoms due to reduced tumor volume and reduced normal brain tissue compression. The other 24 patients were stable throughout treatment and in follow-up.

This study has several potential limitations. First, this is a non-prospective controlled study. Further randomized controlled studies need to be conducted to compare other fractionated radiosurgery approaches with dose-staged Gamma Knife radiosurgery for meningiomas. Second, due to the limited follow-up data of more than 3 years, the longer-term efficacy and safety required full monitoring. Furthermore, the treatment response after Gamma Knife radiosurgery in meningiomas could be influenced by many factors, such as prescription dose, location, volume, pathological classification, and surgical history (35). In this study, no difference was found in the percentage reduction in volume for differential initial volume meningiomas. The outcomes of clinical response and toxicity based on tumor location were illustrated in Table 3, however, among 71 cases, 59 cases (83.1%) were skull base meningiomas, and differential treatment response based on location could not be fully clarified. Future studies to analyze the impact of these prognostic factors on dose-staged Gamma Knife radiosurgery in meningiomas are still needed.

## Conclusion

This study investigates the efficacy and safety of two-stage dose-fractionated Gamma Knife radiosurgery for meningiomas, providing an innovative and minimally invasive treatment option for meningioma patients with contraindications craniotomy. However, optimizing staged radiosurgery and clarifying the radiobiological details relevant to this approach still needed further investigation.

## Data availability statement

The original contributions presented in the study are included in the article/supplementary material, further inquiries can be directed to the corresponding author/s.

## Ethics statement

Ethical review and approval was not required for the study on human participants in accordance with the local legislation and institutional requirements. The patients/participants provided their written informed consent to participate in this study. Written informed consent was obtained from the individual(s) for the publication of any potentially identifiable images or data included in this article.

## Author contributions

Conception and design: JD, EW, LP, and BW. Drafting the article and statistical analysis: XG and JD. Critically revising the article: JK. Approved the final version of the manuscript on behalf of all authors: XG. Study supervision: EW. Analysis and interpretation of data and reviewed submitted version of manuscript: all authors. All authors contributed to the article and approved the submitted version.

## Funding

This study was supported by the National Natural Science Foundation of China grant (No. 81727806) and the Science and Technology Commission of Shanghai Municipality (No. 20194Y0482).

## Conflict of interest

The authors declare that the research was conducted in the absence of any commercial or financial relationships that could be construed as a potential conflict of interest.

## Publisher's note

All claims expressed in this article are solely those of the authors and do not necessarily represent those of their affiliated organizations, or those of the publisher, the editors and the reviewers. Any product that may be evaluated in this article, or claim that may be made by its manufacturer, is not guaranteed or endorsed by the publisher.
